# Participation of miRNA in conditioning the incidence and modification of the course of FAP

**DOI:** 10.1186/1897-4287-9-S2-A7

**Published:** 2011-06-01

**Authors:** A Pławski, M Podralska

**Affiliations:** 1 Institute of Human Genetics, Polish Academy of Sciences, Poznan, Poland

## 

In July 2008, the report considering identification of miRNA associated with regulation of APC protein expression level was published. Two types of MIRNA; miR135a and miR-135b, which are encoded by three genes, were described. Those genes are located on chromosomes 3, 12 and 1. miR135a and miR-135b interact with sequences 3 `UTR of mRNA of gene APC on the basis of incomplete complementarity and lead to its degradation. The changes in the sequences encoding miR135a and miR-135b may lead to a change in their affinity to the 3 `UTR of mRNA of the gene APC and enhancing its degradation. It can result in a reduced number of functional APC protein. Also, changes in the sequence 3 `UTR of mRNA can change the affinity of the sites recognized by miRNA or induce new sites recognized by the miRNA and thus will induce rapid degradation of mRNA. Both these situations would provide a similar effect such as observed in case of the mutations leading to premature termination of protein translation. It could not be excluded that the mutations in the 3 `UTR and miRNA can also modify the disease course if co-occurring with mutations in the APC gene coding sequence.

